# Serum Levels of the Proinflammatory Cytokines IL-6, IL-16, IL-18, and IL-36 in Patients with Multiple Sclerosis from the Western Region of Saudi Arabia: Associations with Disease Duration and Disability

**DOI:** 10.3390/neurolint18090168

**Published:** 2026-09-01

**Authors:** Jehan Alrahimi, Yara Mualla, Alawiah Alhebshi, Hind A. Alnajashi, Kawther Zaher

**Affiliations:** 1 Department of Biological Sciences, Faculty of Sciences, King Abdulaziz University, Jeddah 21589, Saudi Arabia; 2 Immunology Unit, King Fahd Medical Research Center, King Abdulaziz University, Jeddah 21589, Saudi Arabia; 3 Department of Neurology, Faculty of Medicine, King Abdulaziz University, Jeddah 21589, Saudi Arabia; 4 Department of Neurology, King Abdulaziz University Hospital, King Abdulaziz University, Jeddah 21589, Saudi Arabia; 5 Neuroscience and Geoscience Unit, King Fahd Medical Research Center, King Abdulaziz University, Jeddah 21589, Saudi Arabia; 6 Department of Medical Laboratory Sciences, Faculty of Applied Medical Sciences, King Abdulaziz University, Jeddah 21589, Saudi Arabia

**Keywords:** multiple sclerosis, interleukin-6, interleukin-16, interleukin-18, interleukin-36, disability, Expanded Disability Status Scale, Timed 25-Foot Walk, biomarker, Saudi Arabia

## Abstract

**Background:** Systemic inflammation contributes to the pathobiology of multiple sclerosis (MS), yet serum biomarker data from Middle Eastern populations remain limited. This study evaluated serum levels of IL-6, IL-16, IL-18, and IL-36 in Saudi adults with MS compared with matched healthy controls and examined associations with disease duration, phenotype, treatment, and disability. **Methods:** This single-center, cross-sectional, matched case–control study enrolled 26 MS patients and 26 age- and sex-matched controls. Serum cytokines were measured by ELISA. Disability was assessed using EDSS and the Timed 25-Foot Walk (T25FW). Matched comparisons used Wilcoxon signed-rank tests; subgroup comparisons used Wilcoxon rank-sum or Kruskal–Wallis tests; associations used Spearman’s correlation. **Results:** No correction for multiple comparisons was applied given the exploratory design. All four interleukins were higher in MS patients than in controls (*p* < 0.001). Age correlated with EDSS (r = 0.573, *p* = 0.002) and T25FW (r = 0.464, *p* = 0.014). Only IL-6 showed a notable correlation with disability, with T25FW (r = 0.53, *p* = 0.008) and, weaker, EDSS (r = 0.38, *p* = 0.052); IL-18 showed a weak, non-significant correlation with T25FW (r = 0.35, *p* = 0.126); IL-16 and IL-36 showed no relevant associations. IL-16 was higher in newly diagnosed than in long-standing cases (*p* = 0.012); no differences by phenotype or treatment were observed for any marker. **Conclusions:** Serum IL-6, IL-16, IL-18, and IL-36 were elevated in Saudi MS patients versus controls. IL-6 alone showed a modest, unadjusted association with disability, supporting its evaluation as a candidate biomarker in larger, longitudinal, covariate-adjusted cohorts. These findings are hypothesis-generating and should not be interpreted as evidence of independent or clinically actionable biomarker utility.

## 1. Introduction

Multiple sclerosis (MS) is a chronic, immune-mediated, demyelinating, and neurodegenerative disorder of the central nervous system that typically presents in young adults and is a leading cause of non-traumatic neurological disability worldwide. Global prevalence has risen steadily in recent decades [1]. In Saudi Arabia, MS prevalence has been estimated at 40.4 per 100,000 individuals, with disproportionately higher rates reported in the Western Region relative to other parts of the Kingdom, although still below rates observed in some neighboring countries [2,3].

MS pathogenesis reflects an interplay between genetic susceptibility, environmental exposure, and dysregulated innate and adaptive immunity, in which cytokines act as principal effectors and regulators of the balance between demyelinating and reparative processes [4,5]. Associations between circulating or intrathecal interleukin levels and MS disease activity, progression, and disability have already been demonstrated in several cohorts outside the Middle East. Martins et al. reported dysregulated serum cytokine profiles in patients with MS using a multiplexed immunoassay [6]. Kallaur et al. showed that progressive MS is associated with a more pronounced inflammatory imbalance and a greater disability burden than relapsing–remitting disease [7]. Khaibullin et al. reported elevated pro-inflammatory cytokines in the cerebrospinal fluid (CSF) of patients with MS, indicating that inflammatory signaling is present both intrathecally and systemically [8]. Itorralba et al. found that intrathecal IL-6 levels are associated with progressive disease and clinical severity [9]. These studies, however, were conducted in North American and European cohorts; comparatively little work has characterized cytokine profiles in Saudi Arabian or other Middle Eastern populations, despite genetic, environmental, and lifestyle features that distinguish these populations from those in which most cytokine studies have been conducted.

This study focused on four interleukins selected a priori to represent complementary, non-redundant inflammatory mechanisms relevant to MS, rather than an exhaustive interleukin panel. IL-6 is a pleiotropic cytokine involved in acute-phase signaling, Th17 polarization, B-cell activation, and blood–brain barrier disruption, and has previously been linked to MS disease activity and to CSF markers of severity [9,10,11]. IL-16 is a chemoattractant for CD4+ lymphocytes, thought to reflect leukocyte trafficking and sustained immune cell recruitment within inflammatory lesions. Previous work has reported both elevation in MS and a reduction in IL-16 following interferon β-1a therapy [12,13]. IL-18 is an inflammasome-associated, interferon-γ- inducing member of the IL-1 family implicated in innate and adaptive immune amplification and has been reported to be elevated in several independent MS cohorts [14,15,16,17]. IL-36, an IL-1-family cytokine increasingly recognized as a mediator of chronic inflammatory signaling and glial–immune crosstalk, has to date been examined in only one other MS cohort worldwide [5,18]. Because each of these interleukins occupies a distinct position in the inflammatory cascade, we reasoned that evaluating them together, rather than assuming they behave as a single coordinated signal, would provide a more informative picture of which components of systemic inflammation, if any, track with clinical disability; this study was not designed, and was not powered, to test a combined multivariable biomarker model, and no such model is presented.

Clinical assessment of MS disability commonly relies on the Expanded Disability Status Scale (EDSS). This clinician-rated ordinal scale places strong weight on ambulation at higher scores but has recognized limitations, including non-linear scaling and relative insensitivity in some functional domains [19]. The Timed 25-Foot Walk (T25FW) provides a complementary, quantitative, performance-based measure of ambulatory function and is now considered a core outcome measure in MS trials and longitudinal cohorts [20,21]. Linking peripheral interleukin levels to both EDSS and T25FW may help clarify which, if any, aspects of systemic inflammation correspond to clinically meaningful disability.

The present study therefore evaluated serum IL-6, IL-16, IL-18, and IL-36 in patients with MS from the Western Region of Saudi Arabia relative to matched healthy controls, and examined, in an exploratory manner, whether these interleukins, individually or as a broader pattern of inflammatory elevation, were associated with disease duration, clinical phenotype, treatment exposure, and disability as measured by EDSS and T25FW. Given the modest sample size and cross-sectional, hypothesis-generating design, the objective was not to develop, validate, or preferentially foreground any single interleukin as a predefined outcome, but to characterize this exploratory, complementary interleukin panel in an underrepresented population and to report, transparently, whichever associations with clinical disability were and were not observed.

## 2. Materials and Methods

### 2.1. Study Design and Setting

This single-center, cross-sectional, matched case–control study was conducted at the Multiple Sclerosis Clinic of King Abdulaziz University Hospital, Jeddah, Saudi Arabia, from January to June 2024. The study assessed whether serum concentrations of IL-6, IL-16, IL-18, and IL-36 differed between adults with MS and individually matched healthy controls, and whether these interleukins were associated with disability-related measures (EDSS and T25FW) within the MS cohort. All procedures were conducted in accordance with the Declaration of Helsinki.

### 2.2. Ethical Approval and Consent

The study protocol was approved by the Biomedical Ethics Research Committee of King Abdulaziz University, Jeddah, Saudi Arabia (NCBE Registration No. HA-02-J-008; Reference No. 129-24; date of approval: 9 May 2024). Written informed consent was obtained from all participants before enrollment. All data were de-identified before analysis; clinical/demographic data and laboratory results were linked only after completion of measurement and quality-control procedures.

### 2.3. Participants and Eligibility Criteria

A total of 52 adults were enrolled: 26 with clinically confirmed MS and 26 neurologically healthy controls, individually matched 1:1 by sex and within an approximate age range of ±3 years.

MS-group eligibility required ages 18–53 years and a diagnosis established according to the 2017 revised McDonald criteria. The upper age limit was chosen to maintain a relatively homogeneous adult cohort available during the recruitment window, to reduce confounding by older age and age-related comorbidity, and to reflect the age distribution of clinically stable patients seen at the clinic during this period, rather than using a disease-based cutoff. Only clinically stable patients were included, defined operationally as the absence of relapse and no corticosteroid treatment within the 30 days preceding enrollment. Exclusion criteria were: any other central nervous system disorder; systemic autoimmune or inflammatory disease; acute infection; chronic liver or kidney disease; corticosteroid use within the previous 4 weeks; treatment with cytotoxic or other potent immunosuppressive agents; pregnancy; or lactation.

Healthy controls were recruited from hospital staff and community volunteers with no known neurological disease, autoimmune disorder, chronic inflammatory condition, or active infection. They were not receiving corticosteroids or immunomodulatory therapy at the time of sampling.

For each MS participant, the following were recorded: age, sex, disease duration from symptom onset, MS phenotype (relapsing–remitting [RRMS] or secondary progressive [SPMS]), current disease-modifying therapy (DMT), and comorbidities. Disease duration was dichotomized, for exploratory subgroup analysis only, as newly diagnosed (≤2 years from symptom onset) or long-standing (>2 years from symptom onset); this 2-year cutoff was chosen descriptively to separate the cohort at the cohort’s observed median disease duration and does not reflect a validated clinical threshold. DMT exposure was categorized into three mutually exclusive groups: anti-CD20 B-cell-depleting therapy (rituximab, ocrelizumab, or ofatumumab), oral therapy (dimethyl fumarate or teriflunomide), or no current DMT (untreated). No participants in this cohort were receiving injectable platform therapies (interferon-beta or glatiramer acetate) at the time of sampling, so this category does not appear in the analysis.

### 2.4. Clinical Assessment

#### 2.4.1. Expanded Disability Status Scale (EDSS)

Disability was scored by a neurologist experienced in standardized EDSS assessment on a 0–10 scale in 0.5-point increments, performed on the same day as blood collection whenever feasible. The neurologist performing EDSS scoring was blinded to all serum interleukin results, which were generated subsequently as part of batched laboratory analysis [19].

#### 2.4.2. Timed 25-Foot Walk (T25FW)

Participants walked a clearly marked 25-foot (7.62 m) distance as quickly and safely as possible; two trials were completed, and the faster time was used for analysis. Habitual walking aids were permitted and documented.

### 2.5. Blood Collection and Sample Processing

Fasting venous blood (3 mL) was collected between 08:00 and 12:00 to minimize circadian variation in cytokine measurements. Blood was drawn into plain serum tubes, allowed to clot at room temperature for approximately 30 min, then centrifuged at 1500–2000× *g* for 10 min. Serum was aliquoted into labeled polypropylene tubes and stored at −80 °C until analysis. All samples were processed within 1 h of collection and subjected to a single freeze–thaw cycle. Hemolyzed samples were excluded, and visibly lipemic samples were re-collected when feasible.

### 2.6. Cytokine Quantification by ELISA

Serum levels of IL-6, IL-16, IL-18, and IL-36 were measured in duplicate using commercial sandwich enzyme-linked immunosorbent assay kits (BT LAB, Shanghai, China; Cat. Nos. E0090Hu, E0096Hu, E0147Hu, and E7518Hu, respectively) according to the manufacturer’s instructions. Standards and diluted serum samples were added to pre-coated 96-well plates and incubated at 37 °C. Plates were then washed, incubated with biotinylated detection antibody and streptavidin–horseradish peroxidase, and developed with 3,3′,5,5′-tetramethylbenzidine substrate. The reaction was stopped with an acidic stop solution, and optical density was read at 450 nm on a calibrated microplate reader; concentrations were derived from four-parameter logistic standard curves. Samples with duplicate coefficients of variation > 15% were re-assayed, and samples that remained outside acceptance criteria after repeat analysis were excluded from the cytokine-specific analysis. Laboratory personnel were blinded to case–control status, MS phenotype, and treatment exposure throughout.

No serum sample had a missing or non-detectable cytokine concentration; all 52 participants had valid measurements for all four analytes within each assay’s working range. Manufacturer-reported assay characteristics (BT LAB, Shanghai, China) were: IL-6 (Cat. No. E0090Hu), standard curve range 2–600 ng/L, sensitivity 1.03 ng/L; IL-16 (Cat. No. E0096Hu), standard curve range 3–900 ng/L, sensitivity 1.47 ng/L; IL-18 (Cat. No. E0147Hu), standard curve range 0.5–100 ng/L, sensitivity 0.2 ng/L; IL-36 (Cat. No. E7518Hu), standard curve range 18.75–1200 pg/mL, sensitivity 10.13 pg/mL. The lower limit of quantification for each assay corresponded to the lowest point on the standard curve. Samples with a duplicate coefficient of variation exceeding 15% were re-assayed, and all were successfully re-assayed within acceptance criteria; none were excluded from the final analysis.

### 2.7. Statistical Analysis

Analyses were conducted in R (version 3.6.3; R Foundation for Statistical Computing, Vienna, Austria). Distributional characteristics of continuous variables were assessed using histograms, Q–Q plots, and the Shapiro–Wilk test. All continuous variables were non-normally distributed and are reported as medians with interquartile ranges (IQR).

Because healthy controls were individually matched to MS patients by sex and approximate age, the primary case–control comparisons of continuous variables (cytokine concentrations and demographic variables) used the Wilcoxon signed-rank test for paired data. Within the MS cohort, unpaired subgroup comparisons used the Wilcoxon rank-sum test for two-group comparisons (e.g., RRMS vs. SPMS; newly diagnosed vs. long-standing; treated vs. untreated) and the Kruskal–Wallis test for comparisons involving more than two groups (DMT category). Categorical variables were compared using the chi-square test or Fisher’s exact test, as appropriate. Associations between continuous variables (cytokine concentrations, age, EDSS, and T25FW) were assessed using Spearman’s rank correlation coefficient (r), reported with the corresponding coefficient of determination (r^2^) and a two-sided *p*-value; r^2^ is reported alongside r throughout because a coefficient in the moderate range can still leave most of the variance unexplained, and interpreting r together with r^2^ and clinical context has been specifically recommended for correlation coefficients of this magnitude [22].

Every statistical comparison performed in this study, including the variables involved, the groups compared, and the test used, is listed in Supplementary Table S1, ensuring that all analyses, including non-significant results, are fully reported. No post hoc, data-derived cutoffs (e.g., median-split dichotomization of a continuous cytokine) were used anywhere in this study; IL-6 and the other interleukins were analyzed exclusively as continuous variables. Given the exploratory design, modest sample size, and the number of comparisons listn Supplementary Table S1, no formal adjustment for multiple comparisons was applied; consequently, *p*-values throughout this manuscript are reported and should be read as a continuum of evidence from a hypothesis-generating study, not as confirmatory tests of statistical significance. Marginal *p*-values (for example, close to 0.05) are not treated as qualitatively different from *p*-values just above that threshold. A two-sided *p* < 0.05 is used only as a descriptive reference point in the tables below.

## 3. Results

### 3.1. Demographic and Clinical Characteristics

Fifty-two participants were enrolled: 26 MS patients and 26 healthy controls, individually matched 1:1 by sex and approximate age. Median age was 35 years (IQR, 25–42) in MS patients and 33 years (IQR, 25–40) in controls; women comprised 76.9% of each group (Table 1). Smoking status, marital status, education level, and nationality did not differ significantly between groups (Table 1).

### 3.2. Clinical Characteristics of MS Patients

Median disease duration since onset was 2 years (IQR: 1–5). Median BMI was 28.8 kg/m^2^ (IQR, 21.8–30.0); most patients were overweight or obese (65.4%), 30.8% had normal weight, and 3.8% were underweight. A family history of MS was reported by 11.5% of patients. The majority were classified as RRMS (73.1%), with the remainder as SPMS (26.9%). At least one comorbidity was present in 58% of patients. The most common presenting symptoms were numbness (78.8%) and visual weakness (21.1%). On the T25FW, 46.2% of patients completed the walk in ≤4 s, 50% required >4 s, and 3.8% were unable to walk. Regarding treatment, 53.8% of patients received rituximab, 15.4% dimethyl fumarate, 7.7% ocrelizumab, 7.7% teriflunomide, and 3.8% ofatumumab, while 11.5% were untreated at the time of sampli (Table 2).

### 3.3. Serum Cytokine Profile in MS Patients and Matched Controls

**Figure 1:**
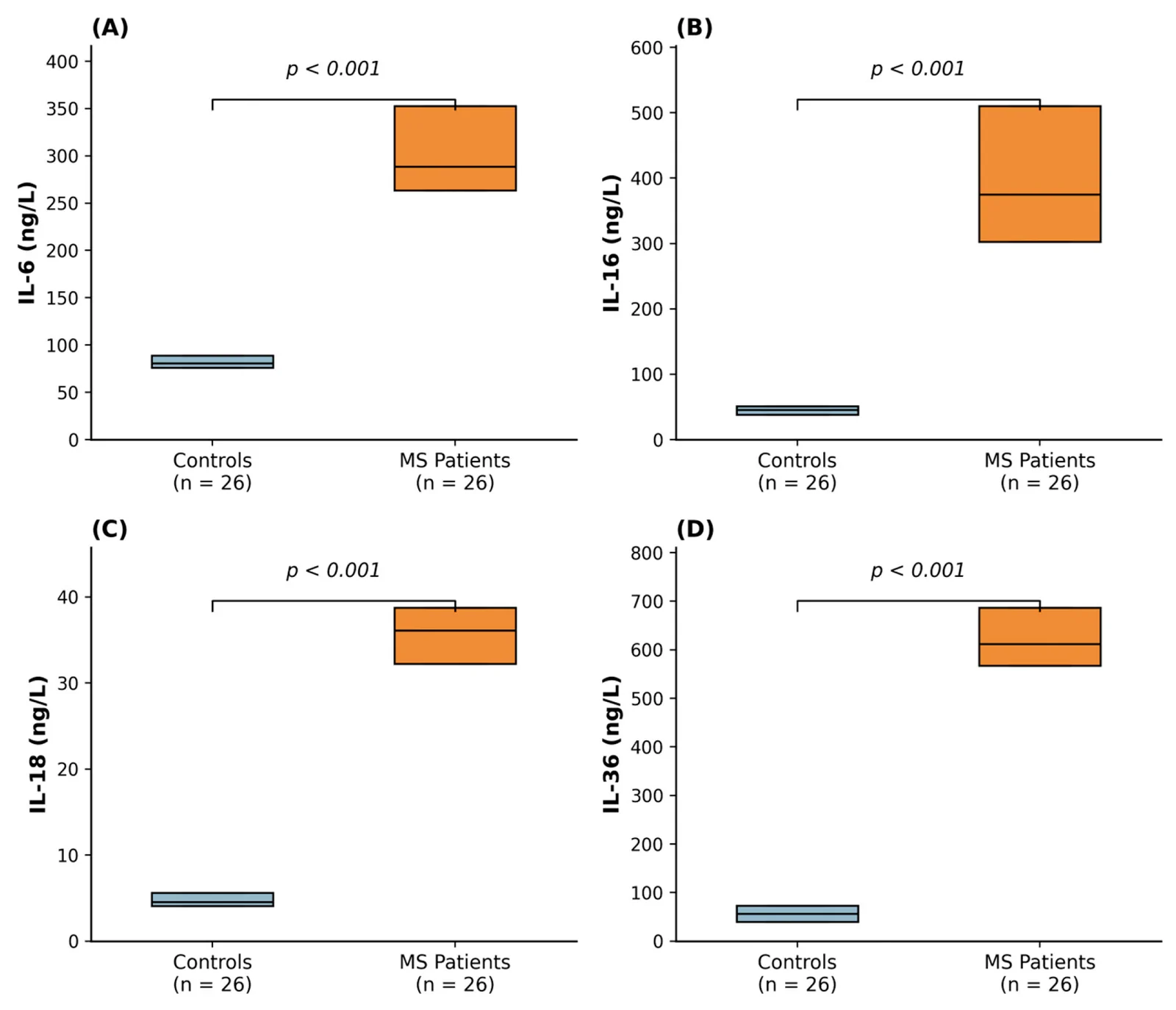
Serum concentrations of (**A**) IL-6, (**B**) IL-16, (**C**) IL-18, and (**D**) IL-36 in matched healthy controls (n = 26) and MS patients (n = 26). Boxes show the median and interquartile range (IQR), as reported n Table 3; the full minimum–maximum range for each group, now available from the retained individual-level measurements, is reported in the paragraph preceding this figure. The whiskers shown here reflect the original Q1–Q3 plot and were not redrawn with the newly retained per-patient values; no individual data points are shown. Group-level separation between MS patients and controls was pronounced for all four interleukins (*p* < 0.001 Table 3; ROC AUC = 1.00 for each, Section Strengths and Limitations). Group comparisons were performed using the Wilcoxon signed-rank test for matched pairs; brackets indicate the case–control comparison for each cytokine—IL, interleukin; MS, multiple sclerosis.

Serum IL-6, IL-16, IL-18, and IL-36 concentrations werh higher in MS patients than in matched controls (Wilcoxon signed-rank test, *p* < 0.001 for all four compariso; Figure 1). Median (IQR) concentrations were: IL-6, 288.52 (263.52–352.74) ng/L in MS patients versus 80.76 (76.02–88.95) ng/L in controls; IL-16, 375.22 (302.54–510.41) ng/L versus 45.59 (38.19–51.15) ng/L; IL-18, 36.11 (32.21–38.77) ng/L versus 4.55 (4.10–5.63) ng/L; and IL-36, 612.00 (567.56–687.00) ng/L versus 56.44 (39.78–73.11) ng/L.

**Table 3:** Serum interleukin concentrations in MS patients and matched controls.

Cytokine	MS Patients, Median (IQR)	Controls, Median (IQR)	*p*
IL-6	288.52 (263.52–352.74) ng/L	80.76 (76.02–88.95) ng/L	*p* < 0.001
IL-16	375.22 (302.54–510.41) ng/L	45.59 (38.19–51.15) ng/L	*p *< 0.001
IL-18	36.11 (32.21–38.77) ng/L	4.55 (4.10–5.63) ng/L	*p *< 0.001
IL-36	612.00 (567.56–687.00) ng/L	56.44 (39.78–73.11) ng/L	*p *< 0.001

Using the individual-level cytokine concentrations subsequently retained, the full observed range (minimum–maximum) for each group was: IL-6, 239.38–656.62 ng/L (MS) vs. 68.69–165.24 ng/L (controls); IL-16, 280.78–669.67 ng/L vs. 32.63–82.63 ng/L; IL-18, 27.40–67.90 ng/L vs. 3.19–8.17 ng/L; IL-36, 395.33–817.56 ng/L vs. 12.00–206.44 ng/L. For every interleukin, the full MS range lies entirely above the full control range, confirming complete, non-overlapping group separation (consistent with the ROC AUC = 1.00 result reported in Section Strengths and Limitations).

### 3.4. Subgroup Comparisons by MS Phenotype, Disease Duration, and Treatment

Table 4 summarizes all within-cohort subgroup comparisons of serum IL-6, IL-16, IL-18, and IL-36. No significant differences in any of the four interleukins were observed between RRMS and SPMS, between DMT categories (anti-CD20, oral, or untreated), or between smokers and non-smokers (all *p* > 0.505 Table 4). The untreated subgroup (n = 3) was too small to support a statistically meaningful two-group comparison against treated patients and was therefore excluded from that specific test (see Section Strengths and Limitations, Limitations); the DMT-category comparison above already includes the untreated group without altering the conclusion. IL-16 concentrations differed significantly by disease duration (*p* = 0.012), with higher levels in newly diagnosed than in long-standing patients; no corresponding difference by disease duration was observed for IL-6, IL-18, or IL-36 (Table 4).

Using the retained individual-level cytokine concentrations, exact subgroup medians (IQR) and a direct anti-CD20 vs. oral comparison are now available. By phenotype: IL-6 was 280.76 (260.93–344.55) ng/L in RRMS (n = 19) vs. 327.31 (297.14–498.86) ng/L in SPMS (n = 7); IL-16 was 375.22 (306.70–495.59) vs. 427.07 (303.93–531.70) ng/L; IL-18 was 36.23 (32.04–39.85) vs. 35.32 (32.95–36.34) ng/L; IL-36 was 589.78 (567.56–664.78) vs. 628.67 (581.44–703.67) ng/L (*p*-values as in the table above). By DMT category (anti-CD20, n = 17; oral, n = 6; untreated, n = 3): IL-6 medians were 287.66, 284.21, and 327.31 ng/L; IL-16 were 375.22, 443.74, and 356.70 ng/L; IL-18 were 36.00, 36.57, and 36.23 ng/L; IL-36 were 606.44, 603.67, and 650.89 ng/L, respectively (*p*-values as in the table above). A direct two-group comparison restricted to anti-CD20 vs. oral therapy, excluding the small untreated group, was then computed directly and confirmed no significant difference for any cytokine (Wilcoxon rank-sum test: IL-6 *p* = 0.944; IL-16 *p* = 0.248; IL-18 *p* = 0.726; IL-36 *p* = 0.674), consistent with the three-group result above.

### 3.5. Correlations Between Age, Interleukins, and Disability-Related Measures

Age correlated positively with both EDSS (r = 0.573, *p* = 0.002) and T25FW (r = 0.464, *p* = 0.014). EDSS and T25FW correlated with each other (r = 0.580, *p* = 0.002). Correlations between age or each interleukin and the two disability-related measures are shown in Table 5, together with the corresponding coefficient of determination (r^2^).

Median EDSS was 1.00 (Q1–Q3, 0.00–2.25) in RRMS versus 3.50 (Q1–Q3, 2.75–4.50) in SPMS patients, a significant difference (*p* = 0.002). Median EDSS did not differ significantly between newly diagnosed and long-standing patients (*p* = 0.096), across DMT categories (anti-CD20 2.50 [1.00–3.00], oral 1.75 [1.12–2.75], untreated 1.00 [0.50–1.50]; *p* = 0.580), or between treated (2.00, Q1–Q3 1.00–3.00) and untreated patients (1.00, Q1–Q3 0.50–1.50; *p* = 0.274).

## 4. Discussion

In this matched case–control study, serum IL-6, IL-16, IL-18, and IL-36 were each elevated in Saudi adults with MS relative to matched healthy controls, consistent with a broad literature showing that peripheral and intrathecal inflammatory mediators are altered in MS [6,7,8,15,16,17,18]. Within the MS cohort, however, the four interleukins did not behave alike with respect to disability: only IL-6 showed a correlation of note, with T25FW (r = 0.53, r^2^ ≈ 0.28) and, more weakly, with EDSS (r = 0.38, r^2^ ≈ 0.14). IL-16 and IL-36 explained less than 3% of the variance in either disability measure, and IL-18 (r = 0.35, *p* = 0.126) was not statistically significant; none of the three is discussed further as a disability correlate below.

This pattern is broadly consistent with and extends prior reports linking IL-6 specifically—rather than a generic interleukin panel—to MS severity. Chen et al. and Eslami et al. reported elevated serum IL-6 in patients with MS, including a possible relationship to disease progression from relapsing–remitting to secondary progressive status [10,11]. Itorralba et al. found that intrathecal IL-6 is associated with progressive disease and clinical severity, although that work measured IL-6 in CSF rather than serum [9]. Guzel et al. and Manouchehrinia et al. likewise reported that EDSS increases with age, consistent with the age–EDSS correlation observed here, and support age as a contributor to the accumulation of disability independent of cytokine levels [23,24]. Bethoux et al. reported a positive relationship between EDSS and T25FW performance, consistent with our finding that the two instruments, though methodologically distinct, capture a shared dimension of overall disease severity [25]. Martins et al., Kallaur et al., and Khaibullin et al. each reported broader panels of dysregulated serum or CSF cytokines in MS, and Berek et al. profiled 65 cytokines and chemokines in paired CSF and serum from patients with MS [6,7,8,26,27]. Takenn together with the present findings, these studies support the general concept that systemic and compartmentalized inflammatory signaling is altered in MS, while our data indicate that, of the four interleukins we measured, this signal tracked with clinical disability only for IL-6.

A plausible explanation for this divergence lies in the distinct signaling roles of these four cytokines. IL-6 is secreted by T cells, B cells, macrophages, microglia, and non-immune cells including endothelial cells and neurons, and plays a pleiotropic role across MS onset and progression [4,28,29]. At the blood–brain barrier, IL-6 is among the pro-inflammatory cytokines produced by resident and endothelial cells that facilitate immune-cell infiltration into the CNS [30,31], and elevated IL-6 in the cerebrospinal fluid has been linked to MS disease activity [29]. IL-6 produced by B cells further promotes Th17 cell differentiation while inhibiting regulatory T-cell generation, and B cells from MS patients exhibit an abnormal pro-inflammatory profile with elevated IL-6 [30]; Th17 cells, in turn, are highly prevalent in active MS lesions [32]. IL-16, by contrast, acts primarily as a CD4+ T-cell chemoattractant reflecting leukocyte trafficking rather than a driver of BBB breakdown or Th17 polarization [33,34]; IL-18 acts largely through the IL-18 receptor/STAT4 pathway to induce IFN-γ [14]; and IL-36 promotes IFN-γ, IL-4, and IL-17 production and dendritic-cell maturation without an established BBB-specific role [5]. This asymmetry in upstream biology—IL-6’s dual role in Th17 polarization and BBB permeability, versus the more circumscribed roles of IL-16, IL-18, and IL-36—offers one candidate explanation for why only IL-1, among the four interleukins measured here, showed a correlation with disability. This explanation is drawn from the broader immunology literature rather than tested directly in this study and remains speculative pending mechanistic follow-up.

IL-16 was elevated in our MS cohort compared with controls, consistent with findings from Nischwitz et al. and Kouchaki et al. [12,13]. IL-16 also differed by disease duration, with higher concentrations in newly diagnosed than in long-standing patients; because this was one of many exploratory subgroup comparisons performed without correction for multiple testing, and our cross-sectional design cannot distinguish a true duration-related pattern from a chance finding in a modest sample, we regard it as hypothesis-generating and recommend confirmation in a longitudinal design. One possible interpretation of higher IL-16 in newly diagnosed than in long-standing disease is a shift from an early phase of active CD4+ T-cell recruitment into CNS lesions, for which IL-16, as a chemoattractant acting through CD4 [33,34,35], would be expected to be relatively elevated, toward a later phase in which sustained antigen exposure and chronic activation lead to a contraction of trafficking-associated signaling, sometimes described as an immune-exhaustion-like state. A broadly analogous temporal pattern has been reported for another interleukin in MS: IL-38 was found at significantly higher serum levels in newly diagnosed than in established MS and systemic sclerosis patients [36,37,38], supporting the general plausibility that interleukin levels track disease stage rather than remain static. However, the mechanisms need not be identical across cytokines. This interpretation is necessarily post hoc, as our cross-sectional design captured a single time point per patient rather than a within-patient trajectory, and should be tested longitudinally before being treated as established. It may also explain why IL-16, unlike IL-6, tracked disease duration but not disability: leukocyte trafficking intensity and cumulative disability accrual are related but not equivalent processes.

IL-18 was elevated in patients relative to controls, consistent with Jahanbani-Ardakani et al., Nicoletti et al., Chen et al., and Losy and Niezgoda [14,15,16,17]. As in Nicoletti et al. and Chen et al., we found no significant correlation between IL-18 and either disability measure [16,17]. IL-36 was likewise elevated relative to controls; to our knowledge, only one other cohort has reported serum IL-36 in MS, similarly finding elevation without a relationship to disease duration, a pattern our data reproduce [18].

Several factors limit how these associations should be interpreted. In this cohort, age correlated with both EDSS and T25FW, and MS phenotype was strongly associated with EDSS (*p* = 0.002). Because no multivariable or partial-correlation analyses were performed, and given the modest overall sample size (post hoc power 48–81% for the IL-6 correlations reported here; Section Strengths and Limitations) and the small SPMS subgroup (n = 7), we cannot determine whether the IL-6–disability association is independent of age, phenotype, or disease duration, or is instead partly or wholly confounded by them. Using the raw ELISA measurements subsequently retained for all 26 MS patients, a partial correlation of IL-6 with EDSS controlling for age and phenotype (RRMS/SPMS) attenuated the association from an unadjusted Pearson r = 0.40 (*p* = 0.043; Spearman r = 0.39, *p* = 0.052) to a partial r = 0.25 (*p* = 0.228, n = 26). In a multivariable linear model (EDSS ~ IL-6 + age + phenotype, R^2^ = 0.56), the IL-6 coefficient was not statistically significant (*p* = 0.248) once age (*p* = 0.036) and phenotype (*p* = 0.014) were included, indicating the unadjusted IL-6–EDSS correlation is at least partly attributable to age and phenotype rather than an independent IL-6 effect. Disease duration could not be added as a covariate: the per-patient duration recomputed from diagnosis dates did not reconcile with the newly diagnosed/long-standing grouping used elsewhere in this study (see Section Strengths and Limitations), so we did not adjust for a duration variable of uncertain provenance. We therefore describe the IL-6 finding as an observed, unadjusted correlation rather than as evidence of an independent biomarker effect. We did not attempt to construct or report a combined interleukin ‘signature’ or ‘profile’: the study measured four individual analytes without composite scoring, clustering, or dimensionality reduction, and only one of them, IL-6, showed a notable correlation with either disability measure. Subgroup comparisons by phenotype, disease duration, DMT category, and treatment status were also based on small and, in the case of treatment, markedly imbalanced groups (17 anti-CD20, 6 oral DMT, 3 untreated); the absence of a significant difference in these comparisons reflects limited statistical power in a modest sample and should not be interpreted as evidence of equivalence or of no underlying biological effect.

From a clinical perspective, serum-based inflammatory markers remain of interest because they are minimally invasive and more feasible for repeated sampling than CSF, particularly where access to advanced imaging or CSF analysis is limited [7,8,26]. However, this cross-sectional, unadjusted, single-center study of 26 patients does not establish diagnostic accuracy, prognostic performance, or readiness for clinical stratification for IL-6 or any other interleukin measured here; at most, these findings support further, larger, and adjusted study of IL-6 as a candidate adjunctive biomarker. Using the individual-level cytokine concentrations subsequently retained, ROC analysis confirmed perfect discrimination between patients and controls for all four interleukins (AUC = 1.00 each; see Limitations), consistent with the pronounced, non-overlapping group separation in Table 3; this reflects strong case–control discriminatory value at the group level and should not be read as evidence of individual-level diagnostic utility in an undiagnosed population.

### Strengths and Limitations

**Figure 2:**
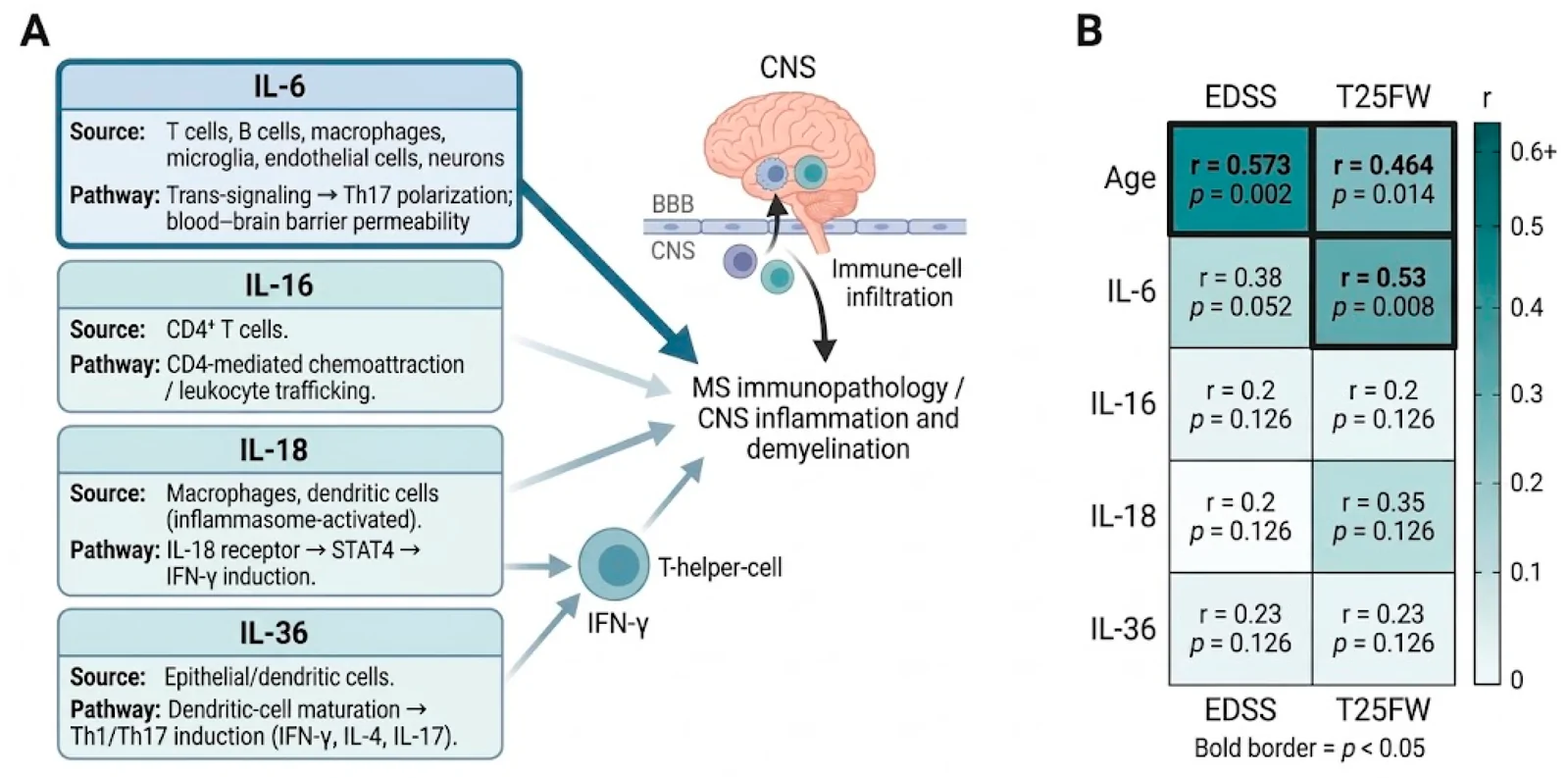
Mechanistic overview and correlation strength of serum interleukins in MS. (**A**) Schematic of the distinct cellular sources and signaling pathways of IL-6, IL-16, IL-18, and IL-36 in MS immunopathology, drawn from the sourced biology summarized in the Discussion above. (**B**) Correlation heatmap of age and each serum interleukin against EDSS and T25FW, built directly from the Spearman r and *p* values already reported in Table 5 (generated from the manuscript’s own values; no new data required).

This study benefits from an individually age- and sex-matched control group, statistical methods that account for that matching, concurrent assessment of two complementary disability measures, and blinding of EDSS scoring and laboratory analysis to case–control status. To our knowledge, it is the second dataset worldwide reporting serum IL-36 in MS. Its principal limitations are as follows:

- Modest sample size, particularly for subgroup comparisons (e.g., n = 7 for SPMS; post hoc power 48–81% for the IL-6 correlations reported here).
- Cross-sectional design, which precludes inferring a causal temporal relationship between cytokine elevation and disability onset and cannot establish whether interleukin elevations precede, accompany, or follow disability.
- Using the individual-level ELISA concentrations subsequently retained, ROC analysis showed perfect discrimination between MS patients and controls for all four interleukins (AUC = 1.00 for IL-6, IL-16, IL-18, and IL-36; Mann–Whitney *p* < 10^−9^ for each), reflecting their fully non-overlapping concentration ranges (Table 3); this is a strong group-level result but does not by itself establish individual diagnostic performance in an undifferentiated clinical population. A partial correlation adjusting the IL-6–EDSS association for age and phenotype was also performed (Discussion, above) and attenuated the association to non-significance (partial r = 0.25, *p* = 0.228); adjustment for disease duration specifically remains a priority for future studies, as the per-patient duration data available to us could not be reconciled with the categorical grouping used elsewhere in this study (Figure 2).
- Lack of correction for multiple comparisons across a substantial number of statistical tests (Supplementary Table S1), which may increase the probability of Type I errors; results should be read as hypothesis-generating rather than confirmatory.
- Use of serum rather than CSF, which may not fully reflect intrathecal inflammatory activity.
- A severe imbalance in treatment-group sample sizes, with anti-CD20 therapy comprising the vast majority of treated patients (17 of 23; 6 oral, 3 untreated), which prevents effective evaluation of the specific impact of individual DMTs on cytokine levels.

This study did not include an a priori sample-size calculation because of its exploratory, hypothesis-generating design. Post hoc power, computed for the Spearman correlations using Fisher’s z-transformation (α = 0.05, two-sided), was approximately 81% to detect the observed IL-6–T25FW correlation (r = 0.53, n = 26), 48% for the IL-6–EDSS correlation (r = 0.38, n = 26), and 42% for the IL-18–T25FW correlation (r = 0.35, n = 26); the study was well powered (≥87%) to detect the age–EDSS correlation (r = 0.573) but only moderately powered (67%) for age–T25FW (r = 0.464). These estimates should be read as approximate, as post hoc power calculations based on an observed effect size are known to be biased and to yield limited estimates of true power; they are reported here to characterize rather than justify the modest sample size. Power for the subgroup comparisons was even lower, particularly for the SPMS subgroup (n = 7) and the untreated subgroup (n = 3), and null results from these comparisons should not be interpreted as evidence of equivalence.

## 5. Conclusions

In this matched case–control study of Saudi adults with MS, serum levels of IL-6, IL-16, IL-18, and IL-36 were elevated relative to those of matched healthy controls. Among these four interleukins, only IL-6 showed a relevant, though modest and unadjusted, correlation with a disability-related measure, most notably T25FW. IL-16 additionally differed by disease duration in this exploratory cohort, a finding that requires longitudinal confirmation. These results provide region-specific, hypothesis-generating evidence on peripheral interleukin biology in an underrepresented Middle Eastern MS population and support prioritizing IL-6s; specifically, for further evaluation in larger, longitudinal, covariate-adjusted studies.

## Figures and Tables

**Table 1:** Baseline demographic characteristics of MS patients (n = 26) and matched healthy controls (n = 26).

Characteristic	MS Patients (n = 26)	Controls (n = 26)	*p*-Value	Test
Age, years, median (IQR)	35 (25–42)	33 (25–40)	0.71	Wilcoxon signed-rank
Female sex, n (%)	20 (76.9)	20 (76.9)	1.00	Matching variable
Current smoker, n (%)	8 (30.8)	7 (26.9)	1.00	Fisher’s exact
Married, n (%)	14 (53.8)	11 (42.3)	0.57	Fisher’s exact
Bachelor’s degree, n (%)	18 (69.2)	14 (53.8)	0.13	Chi-square
Saudi nationality, n (%)	21 (80.8)	26 (100.0)	0.06	Fisher’s exact

**Table 2:** Clinical characteristics of the MS cohort (n = 26).

Characteristic	MS Cohort (n = 26)
Disease duration from onset, years, median (IQR)	2 (1–5)
BMI, kg/m^2^, median (IQR)	28.8 (21.8–30.0)
BMI category, n (%)	Underweight 1 (3.8); Normal 8 (30.8); Overweight 7 (26.9); Obese 10 (38.5)
Family history of MS, n (%)	Yes 3 (11.5); No 23 (88.5)
MS phenotype, n (%)	RRMS 19 (73.1); SPMS 7 (26.9)
≥1 comorbidity, n (%)	Yes 15 (57.7); No 11 (42.3)
T25FW category, n (%)	≤4 s 12 (46.2); >4 s 13 (50.0); Unable to complete 1 (3.8)
Presenting symptoms, n (%)	Numbness 21 (80.8); Visual weakness 6 (23.1)
DMT category, n (%)	Anti-CD20 (rituximab, ocrelizumab, ofatumumab) 17 (65.4); Oral (dimethyl fumarate, teriflunomide) 6 (23.1); Untreated 3 (11.5)

**Table 4:** Subgroup comparisons of serum interleukin concentrations within the MS cohort.

Comparison	IL-6, *p*	IL-16, *p*	IL-18, *p*	IL-36, *p*	Test
RRMS (n = 19) vs. SPMS (n = 7)	0.225	0.563	0.623	0.707	Wilcoxon rank-sum
Newly diagnosed (n = 13) vs. long-standing (n = 13)	0.35–0.86 *	0.012 *	0.09–0.7 *	0.07–0.9 *	Wilcoxon rank-sum
DMT category: anti-CD20 (n = 17) vs. oral (n = 6) vs. untreated (n = 3)	0.644	0.345	0.454	0.557	Kruskal–Wallis
Smokers (n = 8) vs. non-smokers (n = 18)	0.420	0.266	0.344	0.505	Wilcoxon rank-sum

For IL-6, IL-18, and IL-36, only the non-significant result was retained from the original analysis for this comparison; the exact *p*-value was not separately archived. IL-16 was the only cytokine with a significant difference for this comparison (*p* = 0.012), and its exact value is reported in full. * indicates significance (*p* > 0.05).

**Table 5:** Spearman correlations between age, serum interleukins, and disability-related measures in the MS cohort (n = 26).

Variable	vs. EDSS: r (r^2^)	vs. EDSS: *p*	vs. T25FW: r (r^2^)	vs. T25FW: *p*
Age	0.573 (0.33)	0.002	0.464 (0.22)	0.014
IL-6	0.38 (0.14)	0.052	0.53 (0.28)	0.008
IL-16	0.059 (0.003)	0.774	0.16 (0.03)	0.453
IL-18	−0.088 (0.008)	0.669	0.35 (0.12)	0.126
IL-36	−0.019 (<0.001)	0.928	0.09 (0.008)	0.654

## Data Availability

The data presented in this study are available on request from the corresponding author. The data are not publicly available due to institutional privacy restrictions on patient-level clinical data.

## Supplementary Materials

The following supporting information can be downloaded at: https://www.mdpi.com/article/10.3390/neurolint18090168/s1, Table S1: Summary of all statistical analyses performed in this study.

## Abbreviations

The following abbreviations are used in this manuscript:

BMI	Body mass index
CSF	Cerebrospinal fluid
DMT	Disease-modifying therapy
EDSS	Expanded Disability Status Scale
IL	Interleukin
IQR	Interquartile range
MS	Multiple sclerosis
RRMS	Relapsing–remitting multiple sclerosis
SPMS	Secondary progressive multiple sclerosis
T25FW	Timed 25-Foot Walk
Th17	T helper 17
