# Supplementary material for: Serum Levels of the Proinflammatory Cytokines IL-6, IL-16, IL-18, and IL-36 in Patients with Multiple Sclerosis from the Western Region of Saudi Arabia: Associations with Disease Duration and Disability
Source: Neurol Int. 2026 Sep 1;18(9):168. doi: 10.3390/neurolint18090168 (PMC13610939; doi:10.3390/neurolint18090168)
Supplement: Supplementary file 1 [file neurolint-18-00168-s001.zip › neurolint-4502323-supplementary.pdf]

Table S1—summary of all statistical analyses performed in this study.

Analysis	Variable(s)	Groups compared	Statistical test
Baseline continuous variables	Age	MS vs. matched controls	Wilcoxon signed-rank test
Baseline categorical variables	Sex, smoking, marital status, education, nationality	MS vs. matched controls	Chi-square or Fisher's exact test
Primary cytokine comparison	IL-6, IL-16, IL-18, IL-36	MS vs. matched controls	Wilcoxon signed-rank test
MS phenotype subgroup	IL-6, IL-16, IL-18, IL-36	RRMS vs. SPMS	Wilcoxon rank-sum test
Disease-duration subgroup	IL-6, IL-16, IL-18, IL-36	Newly diagnosed vs. long-standing	Wilcoxon rank-sum test
Treatment-status subgroup	IL-6, IL-16, IL-18, IL-36	Treated vs. untreated	Wilcoxon rank-sum test
DMT-category subgroup	IL-6, IL-16, IL-18, IL-36	Anti-CD20 vs. oral vs. untreated	Kruskal–Wallis test
EDSS by phenotype/duration/DMT/treatment	EDSS	As above	Kruskal–Wallis or Wilcoxon rank-sum test
Correlation with disability	IL-6, IL-16, IL-18, IL-36 vs. EDSS	MS cohort only	Spearman's rank correlation
Correlation with gait impairment	IL-6, IL-16, IL-18, IL-36 vs. T25FW	MS cohort only	Spearman's rank correlation
Demographic correlation with disability	Age vs. EDSS; age vs. T25FW	MS cohort only	Spearman's rank correlation
Concordance between disability measures	EDSS vs. T25FW	MS cohort only	Spearman's rank correlation
